# The GlyT1-inhibitor Org 24598 facilitates the alcohol deprivation abolishing and dopamine elevating effects of bupropion + varenicline in rats

**DOI:** 10.1007/s00702-023-02701-x

**Published:** 2023-09-29

**Authors:** Yasmin Olsson, Helga Lidö, Karin Ademar, Davide Cadeddu, Mia Ericson, Bo Söderpalm

**Affiliations:** 1 Addiction Biology Unit, Department of Psychiatry and Neurochemistry, Institute of Neuroscience and Physiology, Sahlgrenska Academy, University of Gothenburg, PO Box 410, 405 30 Gothenburg, SE Sweden; 2 Beroendekliniken, Sahlgrenska University Hospital, Gothenburg, Sweden; 3 Department of Neurology, Sahlgrenska University Hospital, Gothenburg, Sweden

**Keywords:** Dopamine, Glycine receptor, nucleus Accumbens, Ethanol consumption, In vivo microdialysis, Alcohol addiction

## Abstract

Alcohol Use Disorder (AUD) is a relapsing brain disorder that involves perturbations of brain dopamine (DA) systems, and combined treatment with varenicline + bupropion produces additive effects on accumbal DA output and abolishes the alcohol deprivation effect (ADE) in rats. Also, direct and indirect glycine receptor (GlyR) agonists raise basal DA, attenuate alcohol-induced DA release in the nucleus Accumbens (nAc) and reduce alcohol consumption in rats. This study in rats examines whether the GlyT1-inhibitor Org 24598, an indirect GlyR agonist, enhances the ADE-reducing and DA elevating action of the combined administration of varenicline + bupropion in lower doses than previously applied. Effects on voluntary alcohol consumption, the ADE and extracellular levels of glycine and DA in nAc were examined following treatment with Org 24598 6 and 9 mg/kg i.p., bupropion 3.75 mg/kg i.p. and varenicline 1.5 mg/kg s.c., in monotherapy or combined, using a two-bottle, free-choice alcohol consumption paradigm with an ADE paradigm, and in vivo microdialysis in male Wistar rats. Notably, all treatment regimens appeared to abolish the ADE but only the effect produced by the triple combination (Org24598 + varenicline + bupropion) was significant compared to vehicle. Hence, addition of Org 24598 may enhance the ADE-reducing action of varenicline + bupropion and appears to allow for a dose reduction of bupropion. Treatment with Org 24598 raised accumbal glycine levels but did not significantly alter DA output in monotherapy. Varenicline + bupropion produced a substantial elevation in accumbal DA output that was slightly enhanced following addition of Org 24598. Conceivably, the blockade of the ADE is achieved by the triple combination enhancing accumbal DA transmission in complementary ways, thereby alleviating a hypothesized hypodopaminergia and negative reinforcement to drink. Ultimately, combining an indirect or direct GlyR agonist with varenicline + bupropion may constitute a new pharmacological treatment principle for AUD, although further refinement in dosing and evaluation of other glycinergic compounds are warranted.

**Supplementary Information:**

The online version contains supplementary material available at 10.1007/s00702-023-02701-x.

## Introduction

Alcohol Use Disorder (AUD) is a chronically relapsing brain disorder that involves adaptations in the brain reward circuitry and is characterized by an impaired ability to control alcohol intake despite adverse consequences (Hasin et al. 2013; Koob and Volkow 2016). Worldwide, 5% of all deaths and disability-adjusted-life-years (DALYs) are attributable to alcohol, also classified as a main, yet preventable, contributor to the global burden of disease (Rehm et al. 2017). Moreover, the risk of experiencing alcohol-related harm corresponds to the average amount of alcohol consumed (Rehm et al. 2017). AUD involves high alcohol consumption and thus generates enormous harm for afflicted individuals and the society at large (Rehm et al. 2017). Pharmacological interventions are important in combating AUD, but available medications present small effect sizes and improved pharmacotherapies are warranted (Maisel et al. 2013; Jonas et al. 2014). Presumably, improved treatment outcomes could be achieved by specifically targeting the pathophysiology of AUD and/or mechanisms by which alcohol interferes with the brain reward system.

A large body of evidence suggests that alcohol’s positively reinforcing properties entail enhanced dopamine (DA) activity in the nucleus Accumbens (nAc), a central component of the mesolimbic DA system (Di Chiara and Imperato 1988). In addition, neuroadaptations in these circuits are involved in the transition from recreational to compulsive drinking characteristic for AUD, and in the emergence of a hypodopaminergic state (Koob and Volkow 2016). The hypodopaminergic state is widely recognized to be a neuronal correlate for dysphoria and a driving force for renewed alcohol intake by negative reinforcement (Koob and Volkow 2016), although conflicting results on basal DA levels following protracted abstinence have recently been presented (Hansson et al. 2019). Moreover, it is still unclear exactly how alcohol acts on the mesolimbic DA pathway on a molecular and cellular level.

Interestingly, data obtained from several previous studies suggest that alcohol acts on inhibitory glycine receptors (GlyR) in the nAc, that inhibit γ-aminobutyric acid (GABA)-ergic, medium spiny neurons (MSNs) projecting to the ventral tegmental area (VTA), ultimately resulting in enhanced DA release in the nAc (Molander and Söderpalm 2005b; Forstera et al. 2017; Soderpalm et al. 2017). Moreover, perfusion of glycine into the nAc or systemic treatment with glycine-transporter-1(GlyT-1)-inhibitors, elevates DA levels, attenuates alcohol-induced DA elevation and reduces alcohol intake, with no signs of tolerance development, in the rat (Molander and Söderpalm 2005b; Molander et al. 2007; Lidö et al. 2009, 2012; Vengeliene et al. 2018). GlyT1 is predominantly expressed on glial cells, but also on postsynaptic glutamatergic neurons, and regulates central nervous system (CNS) glycine levels together with GlyT2 (Cioffi 2021). Previous studies show that GlyT1-inhibitors, *e.g.* Org 24598 (sarcosine-based), as compared to direct GlyR agonists, are the most efficient glycinergic agents available for reducing alcohol intake, presumably by potentiating GlyR neurotransmission (Lidö et al. 2012; Vengeliene et al. 2018; Olsson et al. 2020, 2022; Ulenius et al. 2020). Conceivably, enhanced GlyR signaling targets the positive reinforcing properties of alcohol by attenuating the alcohol-induced DA elevation while simultaneously mitigating negative reinforcement, *i.e.* hypodopaminergia and associated anhedonia, by raising basal nAc DA levels. Consequently, potentiation of GlyR signaling in the CNS may constitute a new mechanism for interfering with the reinforcing properties of alcohol and ultimately a new pharmacologic treatment principle for AUD (Molander et al. 2005; Harvey and Yee 2013; Forstera et al. 2017; Soderpalm et al. 2017).

Nonetheless, alcohol directly or indirectly interferes with several neurotransmitter systems besides glycine, *e.g.* acetylcholine (ACh) and noradrenaline signaling systems among others (Ericson et al. 2003; Larsson et al. 2005; Miller and Kamens 2020; Vena et al. 2020; Loftén et al. 2021). It is conceivable that potentiated GlyR signaling alone might not be sufficient to completely block the positive reinforcing properties of alcohol and/or sufficiently counteract the hypodopaminergic state present in the addicted brain, as underlined by the lack of benefit for a GlyT1-inhibitor over placebo in preventing relapse in AUD patients (de Bejczy et al. 2014). Instead, pharmacotherapy directed at more than one target might prove advantageous and is currently examined in an ongoing clinical trial combining bupropion, a noradrenaline- and DA reuptake inhibitor, and varenicline, a partial nicotinic acetylcholine receptor (nAChR) agonist (*cf.* clinicaltrials.gov, NCT 04167306). Varenicline has previously been shown to reduce alcohol intake in rats and humans (Steensland et al. 2007; McKee et al. 2009; Litten et al. 2013; de Bejczy et al. 2015). Interestingly, accumbal DA levels are raised following treatment with either varenicline or bupropion and additive effects (a twofold increase in extracellular DA levels) are produced following combined treatment in the rat (Söderpalm et al. 2020). Moreover, the combination treatment completely blocks the alcohol deprivation effect (ADE), an animal model ascribed a high predictive value since approved medications to treat AUD (*i.e.* acamprosate and naltrexone) also abolish this effect (Spanagel 2009; Maisel et al. 2013; Söderpalm et al. 2020). The favorable outcome may be explained by that bupropion and varenicline interact with the mesolimbic reward circuit and raise extracellular accumbal DA levels in vivo through two separate mechanisms, *i.e.* by stimulating (presumably tonic) DA release and by inhibiting DA reuptake (Söderpalm et al. 2020).

**Fig. 1:**
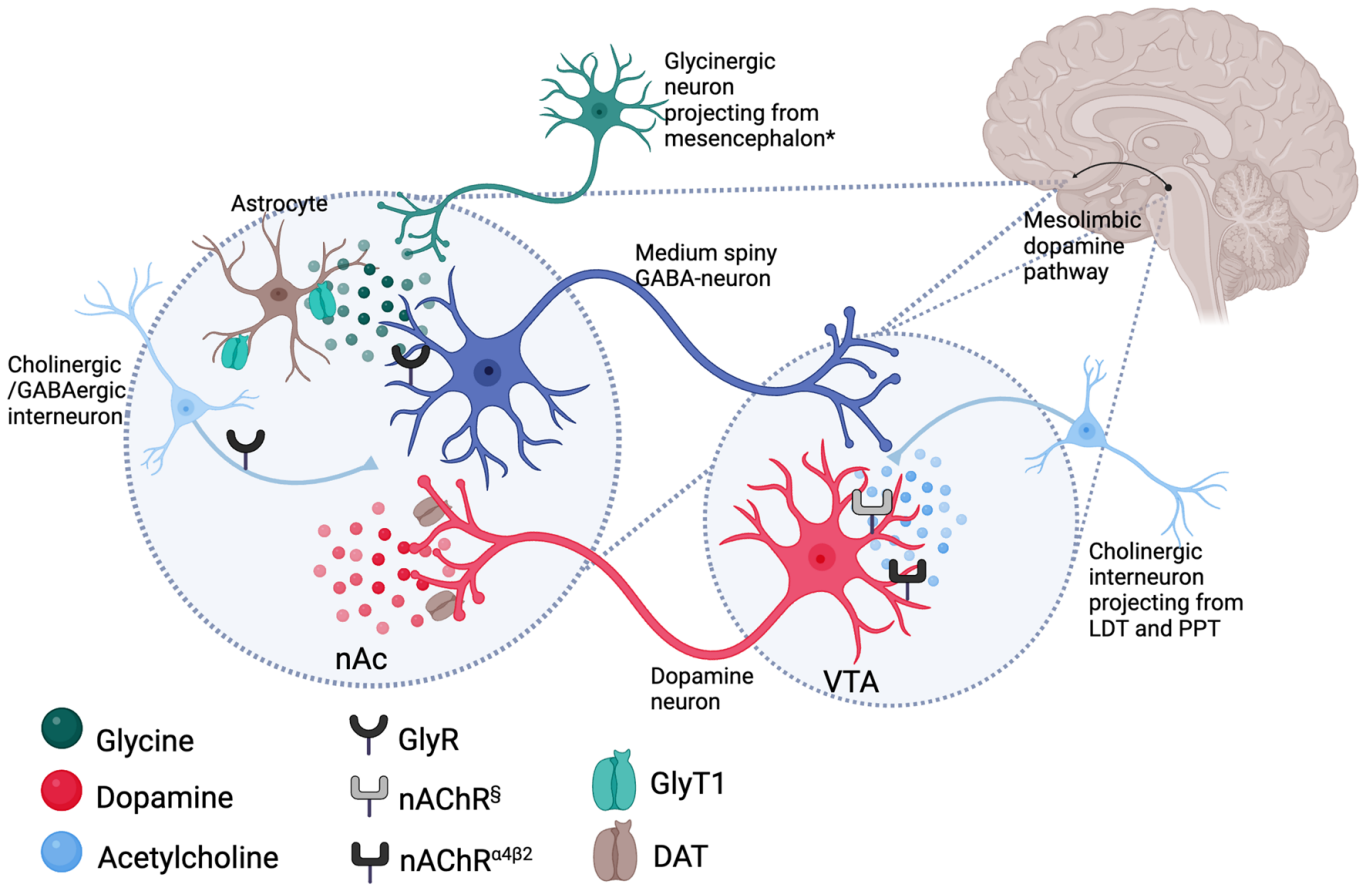
Schematic overview of hypothesized molecular and cellular effects of the triple combination treatment on the mesolimbic dopamine reward system. The glycine transporter-1(GlyT-1)-inhibitor Org 24598 inhibits GlyT1, expressed on glial cells, and raises extracellular levels of the endogenous glycine receptor (GlyR) agonist glycine in the nucleus Accumbens (nAc). Consequently, inhibitory GlyRs presumably located on γ-aminobutyric acid (GABA)-ergic spiny projection neurons and/or GABAergic or cholinergic interneurons are potentiated, thereby inhibiting GABA-ergic projections to the ventral tegmental area (VTA). Ultimately, tegmental dopaminergic firing, that involves activation of nicotinic acetylcholine receptors (nAChRs), different from α4β2 nAChRs, is disinhibited whereby nAc dopamine (DA) levels are raised. Varenicline is instead thought to act on α4β2 nAChRs present on tegmental DA neurons, whereas bupropion raises nAc DA levels by inhibiting DA transporters (DAT) on DA terminals. Consequently, the triple combination treatment interferes with mesolimbic DA signaling in three complementary ways. lPAG, lateral periaqueductal gray; LDT, laterodorsal tegmental nucleus; PPT, pedunculopontine tegmental nucleus, ^§^ nAChRs different from α4β2 nAChRs, *(Konar 2022). The figure was created with BioRender.com.

Conceivably, adding a third agent that targets the DA reward system via yet another mechanism, *i.e.*, by means of raising central glycine levels to enhance GlyR signaling using a GlyT1-inhibitor, may yield further, additive or synergistic effect in restoring accumbal DA levels and/or reducing alcohol consumption (Fig. 1). This could also allow for dose reductions, which may mitigate side effects recognized for bupropion and varenicline, *e.g.* insomnia, headache and nausea, thereby improving patient adherence (Anthenelli et al. 2016). Hence, the aim of this study was to examine the effects of combined treatment with threshold doses for Org 24598, bupropion and varenicline on voluntary alcohol intake and accumbal DA levels in rats.

## Materials and methods

### Animals

**Fig. 2:**
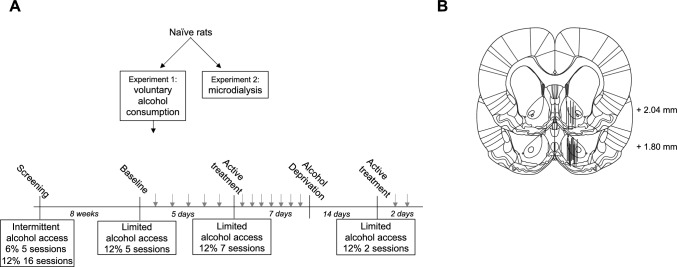
Overview of experimental design and probe placement verification. **A** Schematic overview of the experimental design. In a first set of experiments, effects on voluntary alcohol intake and the alcohol deprivation effect were evaluated. Next, effects on accumbal glycine and dopamine levels were examined using in vivo microdialysis in a new group of rats. Grey arrows indicate systemic injections with vehicle at baseline and experimental drugs during the active treatment phase. **B** Location of 17 representative microdialysis probes in the nAc. Black bars indicate the active space of the probe and targets the nAc core–shell borderline region. Distance from bregma is indicated on the right side of the figure

A schematic overview of all experiments is provided in Fig. 2A. For the alcohol consumption study, 64 male Wistar rats weighing 160–180g upon arrival were supplied by Envigo (Netherlands). During the first week, rats were housed four to a cage under controlled environmental conditions that comprised regular light–dark conditions with lights on at 07:00 a.m. and off at 07:00 p.m, constant room temperature of 20–22 °C and humidity of 50–65%. Thereafter, rats were single-housed with a reversed light–dark-cycle, *i.e.* lights off at 10:00 a.m. and lights on 10:00 p.m. For in vivo microdialysis, a total number of 40 male Wistar rats weighing 260–280g upon arrival were supplied by Envigo (Netherlands). Rats were housed four to a cage prior to surgery and single-housed after surgery under controlled environmental conditions with a regular light–dark-cycle as above. Before initiation of experimental procedures, all animals were allowed at least one week of acclimatization to the animal facility. All rats had free access to standard rat feed (Haklan Teklad, England) and tap water. The experiments were approved by the Ethics Committee for Animal Experiments, Gothenburg, Sweden (ethical approval reference number 2401-19 and 3095-20).

### Drugs

Org 24598 (Tocris, England) was dissolved in 0.01M phosphate-buffered saline (PBS) (Sigma-Aldrich, Sweden) to a desired threshold dose of 6 or 9 mg/kg and administered i.p. in a volume of 5 ml/kg, *i.e.* a lower dose than the previously studied dose of 12 mg/kg which reduced alcohol intake without tolerance development and produced higher accumbal glycine levels as compared to treatment with PBS in rats (Lidö et al. 2012). Bupropion (Sigma-Aldrich, Sweden) was dissolved in PBS to a desired threshold dose of 3.75 mg/kg and administered i.p. in a volume of 5 ml/kg. Varenicline benzazepine tartrate, kindly provided by Pfizer Global Research, was dissolved in 0.9% saline to a desired dose of 1.5 mg/kg and administered s.c. in a volume of 2 ml/kg. The doses for bupropion and varenicline were chosen based on a previous study in which bupropion 5 mg/kg, *i.e*. a higher dose than applied in the present study, and varenicline 1.5 mg/kg blocked the ADE and produced additive effects on accumbal DA levels, whereas bupropion 2.5 mg/kg and varenicline 1.5 mg/kg did not (Söderpalm et al. 2020). Rats in all treatment groups, including the vehicle group that was treated with PBS i.p. and NaCl s.c., received equal amounts of fluids and total number of injections. In order to keep the injection volumes at a minimum, Org 24598 and bupropion were administered in one injection. Ethanol (95%; Kemetyl AB, Sweden) was diluted in tap water to a concentration of 6 and 12% (v/v), also in line with alcohol concentrations chosen in the previous study for combined treatment with bupropion and varenicline (Söderpalm et al. 2020).

### Voluntary alcohol consumption study

**Fig. 3:**
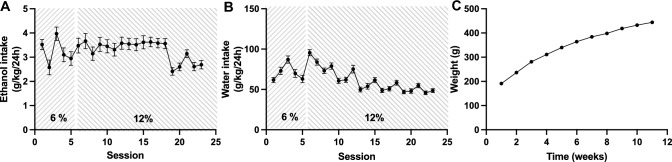
Screening for voluntary alcohol intake and weight gain.** A** Alcohol and **B** water intake remained on a stable level throughout the screening phase for alcohol intake. **C** Rats presented a stable weight gain throughout the entire experiment. During screening, rats had intermittent access to alcohol (three 24h-sessions/week). Shown are mean values ± SEM

Throughout the experiment (Fig. 2A), all rats had free access to tap water and standard rat feed, were monitored daily and weighed each week (Fig. 3C). During the first eight weeks, rats were screened for alcohol intake and had intermittent (3 sessions/week) access to alcohol, while alcohol (Fig. 3A), and water intake (Fig. 3B) were monitored. Rats were presented with an alcohol concentration of 6% (v/v) during the first five sessions followed by 12% (v/v) for the remainder of the experiment. The alcohol bottles were added concurrent with the beginning of the dark period and removed after 24 h. Following the initial screening phase, rats with an alcohol intake exceeding 1.5 g EtOH/kg/day were placed on a limited access paradigm that comprised access to 12% (v/v) alcohol for 8 h/day. During 5 days of baseline monitoring, rats received preparatory vehicle injections in volumes equal to active treatment in order to minimize the influence of stress brought by the injection on alcohol consumption. Thereafter, 56 rats were divided into four treatment groups based on their average alcohol consumption and received either active treatment (Org 24598; bupropion and varenicline; Org 24598, bupropion and varenicline) or vehicle for seven consecutive days. All injections were administered 30 min prior to access to alcohol and water, in similarity to a previously performed study on bupropion and varenicline (Söderpalm et al. 2020). Following the active treatment phase, rats were deprived from alcohol for 14 days, whereafter limited access to alcohol and active treatment were re-instated for another 2 days. Attenuation of the ADE has a high predictive validity for clinical effect and the model itself has previously been ascribed to reflect a modality of AUD, *i.e.* loss of control (Spanagel 2009).

### Microdialysis study

Probe placement surgery was performed 2 days prior to the microdialysis experiment. The rats were anesthetized with 4% isoflurane (Baxter, Sweden) and mounted onto a stereotactic instrument (David Kopf Instruments, USA). In order to prevent hypothermia and dehydration and provide analgesia during surgery, rats were placed on a heating pad, received 2 ml 0.9% saline and 1 mg/kg Metacam (Boehringer Ingelheim, Sweden) s.c and Marcain (bupivacaine, AstraZeneca, Sweden) infiltrated along the surgical incision. Three holes were drilled in the skull for the placement of a probe and two anchoring screws. An I-shaped, custom-made probe with a semi-permeable membrane (cut-off 20 kDa) and an active space comprising 2 mm was gently lowered into the nAc core–shell borderline region (A/P: + 1.85, M/L: − 1.4, D/V: − 7.8 mm relative to bregma and dura), coordinates from Paxinos and Watson 2007. This sub-region of the nAc has previously been associated with alcohol-induced DA elevation (Howard et al. 2009). Harvard cement (Dental AB, Sweden) was used to fix the probe and anchoring screws to the skull.

At the day of the experiment, rats were connected to a microinfusion pump (U-864 Syringe Pump, AgnTho’s, Sweden) via a swivel, allowing the animal to move around freely. Prior to sampling, the probes were perfused with Ringer’s solution during 2 hours in order to achieve equilibrium between the perfusate and extracellular fluid. The infusion rate was set at 2 μl/min and dialysate samples (40 μl) were collected every 20 min. The first three consecutive DA samples constituted the baseline and was set to 100% for each animal. Animals with fluctuations at baseline exceeding 10% were excluded. Following administration of designated treatments, dialysate samples were collected for another 140 min. DA samples were analysed online and the remainder of the dialysate was preserved in sodium azide and stored at a cold temperature for later analyses of glycine content. The dialysate DA and glycine content were separated and quantified using a high-performance liquid chromatography (HPLC) system coupled to electrochemical (DA) or fluorescent (glycine) detection as previously described (Ulenius et al. 2020). External standards containing 3.25 nM of DA versus 500 and 1000 nM of glycine were used for identification of peaks in the chromatogram and quantification of respective analyte. At the end of the experiment, rats were sacrificed, and brains were cut into thin slices using a vibroslicer (Campden Instruments Ltd., USA) for verification of correct probe placement using the naked eye. Rats with incorrect probe placement or substantial hemorrhage were excluded. Probe placement is illustrated in Fig. 2B.

### Statistics

A Shapiro–Wilk’s-test > 0.05 and a visual inspection of Q–Q-plots indicated that data within groups were normally distributed, whereas a Brown–Forsythe’s test > 0.05 indicated equal variances across groups. Alterations in alcohol consumption and microdialysate content over time were evaluated by a two-way analysis of variance (ANOVA) with repeated measures, whereas comparisons of area under the curve (AUC) data were performed using a one-way ANOVA, followed by Tukey’s post-hoc when applicable. Alcohol intake prior to and after the alcohol deprivation period was evaluated with a paired *t*-test for effects within a treatment group and with a one-way ANOVA for effects between different treatment groups. All data are presented as mean ± SEM. A probability value (*p*) < 0.05 was considered statistically significant. Statistical analyses were performed using GraphPad Prism version 9.3.1 for Mac OS (GraphPad Software Inc., USA).

## Results

### Effects of Org 24598, bupropion and varenicline on voluntary alcohol intake

**Fig. 4:**
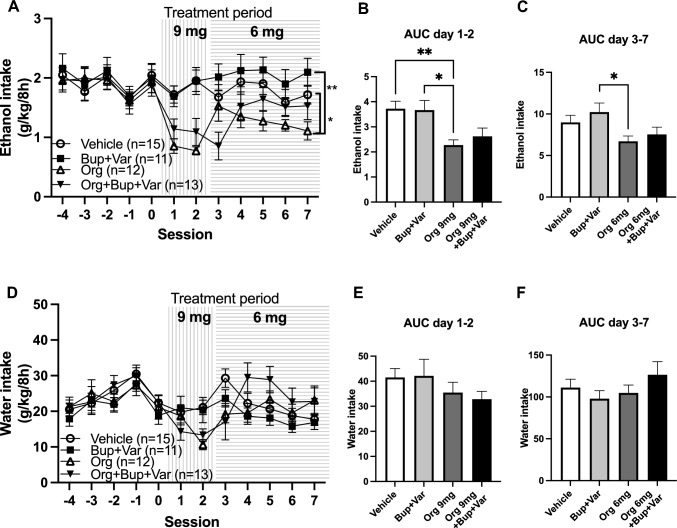
Effects of Org 24598 6 and 9 mg/kg, bupropion and varenicline, alone or combined, on voluntary alcohol and water intake. **A** Alcohol intake over time following 7 days of treatment with vehicle, bupropion + varenicline or Org 24598 alone or combined with bupropion + varenicline. Rats treated with Org 24598 received a higher dose (9 mg/kg) during the first 2 days, whereafter it was reduced to 6 mg/kg for the remaining 5 days of active treatment. **B**, **C** Comparisons of AUCs between groups for day 1–2 and day 3–7 revealed a reduced alcohol intake following treatment with the higher dose (9 mg/kg) of Org 24598 as compared to vehicle injections. Moreover, both doses of Org 24598 reduced alcohol intake as compared to combined treatment with bupropion + varenicline. **D**–**F** Water intake was not significantly altered following any of the designated treatment regimens. During baseline and the treatment period, rats had limited access to alcohol (8h-sessions/day). Shown are mean values ± SEM, *n* = number of rats, *p < 0.05, **p < 0.01

In a first set of experiments, rats were screened for voluntary alcohol intake. As pictured in Fig. 3A, B alcohol and water intake remained relatively stable throughout the screening period with the average daily alcohol intake over time being 3–4 g/kg/day. Animal weight was monitored on a weekly basis throughout the entire experiment and a stable weight gain was observed (Fig. 3C). Following eight consecutive weeks of intermittent access to alcohol, rats with an ethanol intake above 1.5 g/kg/day were put on a limited alcohol access paradigm with preceding, daily vehicle injections, constituting 5 days of baseline. This group of rats had a mean daily alcohol intake of 3.6 g/kg/day and are likely to present with a relative extracellular hypodopaminergia in the nAc (Ericson et al. 2020). Thereafter, the rats were divided into four treatment groups (Org 24598 9 mg/kg; Org 24598 9 mg/kg + bupropion + varenicline; bupropion + varenicline; vehicle), that presented equal alcohol intake. On the second day of active treatment, slight motor deficiencies combined with a non-significant trend for reduction in water intake (Fig. 4D) were observed for animals treated with Org 24598, thus motivating a dose reduction from 9 to 6 mg/kg. Treatment with Org 24598 (day 1–2: 9 mg/kg; day 3–7: 6 mg/kg) reduced alcohol intake as compared to treatment with vehicle or bupropion + varenicline (treatment effect *F*_(3,47)_ = 4.68, *p* = 0.0061, time effect *F*_(7,329)_ = 8.31 *p* < 0.0001, interaction term *F*_(21,329)_ = 2.49, *p* = 0.0004, Org 24598 9mg/kg vs vehicle *p* = 0.045, Org 24598 9mg/kg vs bupropion + varenicline *p* = 0.01) (Fig. 4A), but did not significantly alter water intake (Fig. 4D). In order to differentiate the effects for the higher vs lower dose of Org 24598, AUCs for day 1–2 and 3–7 were analyzed separately. Indeed, the higher dose of Org 24598 reduced alcohol intake as compared to treatment with vehicle or bupropion + varenicline (treatment effect *F*_(3,47)_ = 5.47, *p* = 0.026, Org 24598 vs vehicle *p* = 0.0085, Org 24598 vs bupropion + varenicline *p* = 0.0227) (Fig. 4B), whereas the lower dose produced a significant reduction only when compared to rats receiving bupropion + varenicline (treatment effect *F*_(3,47)_ = 2.91, *p* = 0.0444, Org 24598 vs bupropion + varenicline *p* = 0.0475) (Fig. 4C). Preference for alcohol was not significantly altered following any of the designated treatment regimens (Supplementary Fig. 1.) Moreover, neither treatment with bupropion + varenicline nor with Org 24598 + bupropion + varenicline produced any significant effect on alcohol intake as compared to vehicle. Water intake (Fig. 4D–F) was not significantly altered by any treatment regimens.

### Effects of Org 24598, bupropion and varenicline on the alcohol deprivation effect

**Fig. 5:**
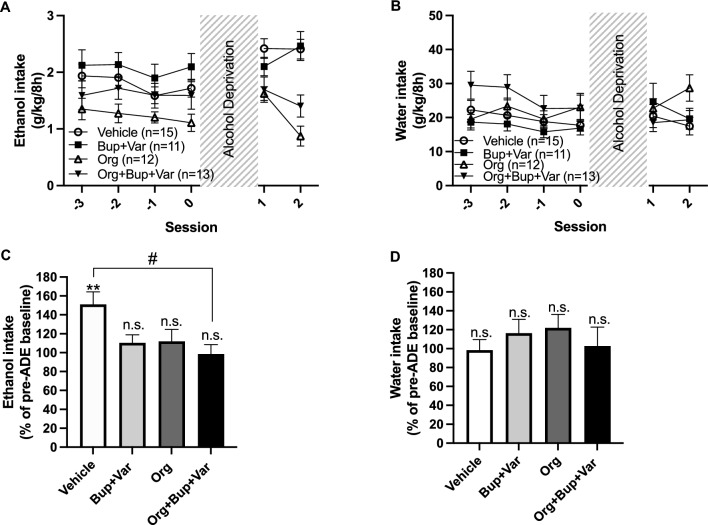
Effects of Org 24598 6 and 9 mg/kg, bupropion and varenicline, alone or combined, on the alcohol deprivation effect (ADE). Time course graph depicting **A** alcohol intake and **B** water intake prior to and 2 days after the 2-week-long alcohol deprivation period. **C** Rats in the vehicle-treated group presented an ADE, *i.e.* an increased alcohol intake (mean of 2 days post alcohol deprivation) as compared to their average alcohol consumption during three consecutive days prior to the alcohol deprivation period. In contrast, the ADE was blocked in the other treatment groups (Org 24598 6 and 9 mg/kg; bupropion + varenicline; Org 24598 6 and 9 mg/kg + bupropion + varenicline). Upon comparison between groups of change in alcohol intake prior to and after the alcohol deprivation period, only the triple combinatory treatment significantly differed from treatment with vehicle. **D** Water intake was not altered prior to and after the alcohol deprivation period. During the two treatment days that followed the alcohol deprivation period, rats had limited access to alcohol (8h-sessions/day). Shown are mean values ± SEM, *n* = number of rats, *p < 0.05, **p < 0.01, significant compared with baseline, #*p* < 0.05, significant as compared with vehicle-treated control

Following 7 days of active treatment, rats were put on an alcohol deprivation paradigm for 14 days. Thereafter, active treatment and limited access to alcohol were re-introduced for two consecutive days and alcohol (Fig. 5A) and water intake (Fig. 5B) prior to (mean of three last values) and after the alcohol deprivation period (mean of two values) were compared. As depicted in Fig. 5C, vehicle-treated rats presented an ADE, *i.e*. an increased alcohol consumption following the alcohol deprivation period, whereas the ADE was blocked in all other treatment groups (*t*_(14)_ = 3.90, *p* = 0.0016). After a between groups comparison of the relative change in alcohol intake (Fig. 5C) prior to and after the alcohol deprivation period, only the triple combinatory treatment significantly differed from vehicle (treatment effect *F*_(3,45)_ = 4.14, *p* = 0.0113, vehicle vs Org 24598 + bupropion + varenicline *p* = 0.0114), whereas water intake was not altered (Fig. 5D). During the 2 days of active treatment that followed the alcohol deprivation period, alcohol and water intake were stable within groups, except for rats receiving Org 24598 alone that further reduced their alcohol intake on the second day (*t*_(11)_ = 4.74, *p* = 0.0006).

### Effects of Org 24598 6 mg/kg, bupropion and varenicline, alone or combined, on accumbal glycine and dopamine levels

**Fig. 6:**
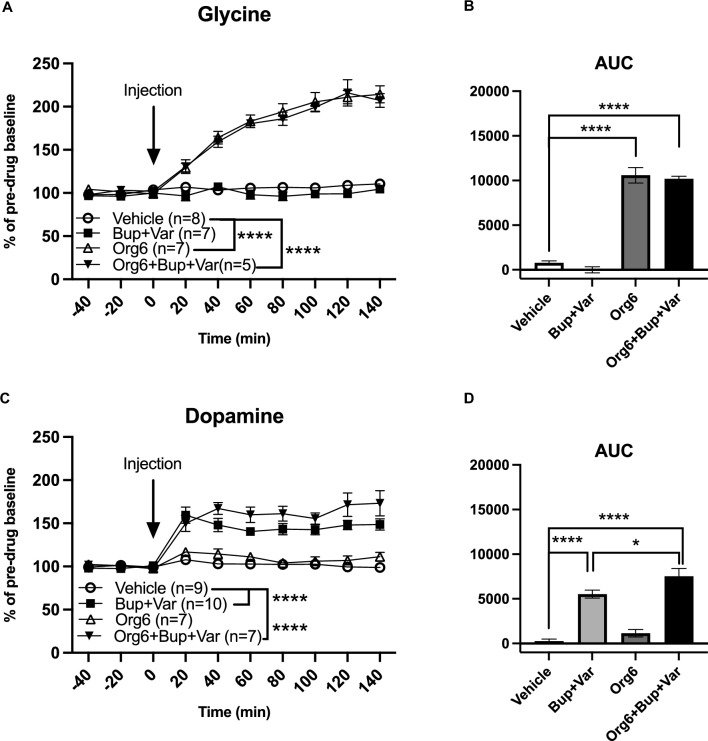
Effects of Org 24598 6 mg/kg, bupropion and varenicline, alone or combined, on accumbal glycine and dopamine (DA) levels. **A**, **B** Systemic treatment with Org 24598 6 mg/kg alone and combined with bupropion + varenicline raised accumbal glycine levels, whereas the combination of bupropion and varenicline did not alter accumbal glycine. **C**, **D** Accumbal DA levels were raised following treatment with bupropion and varenicline with or without the addition of Org 24598 6 mg/kg. The triple combinatory treatment presented significantly higher DA output as compared to the double combinatory treatment, upon comparisons of AUCs. Shown are mean values ± SEM, *n* = number of rats, *p < 0.05, ****p < 0.0001

Finally, the lower dose of Org 24598 was evaluated in the microdialysis model. Treatment with Org 24598 6 mg/kg, administered alone or combined with bupropion and varenicline, raised accumbal glycine levels in a progressive and sustained manner when evaluating effects over time (treatment effect *F*_(3,23)_ = 124.6, *p* < 0.0001, time effect *F*_(6,138)_ = 41.0, *p* < 0.0001, interaction term *F*_(18,138)_ = 12.8, Org 24598 6 mg/kg vs vehicle, *p* < 0.0001, Org 24598 6mg/kg + bupropion + varenicline vs vehicle *p* < 0.0001, Fig. 6A) and upon comparison of AUCs (*F*_(3,23)_ = 127.2, *p* < 0.0001, Org 24598 6mg/kg vs vehicle, *p* < 0.0001, Org 24598 6mg/kg + bupropion + varenicline vs vehicle *p* < 0.0001, Fig. 6B). Accumbal DA levels were not significantly altered following treatment with Org 24598 6 mg/kg (Fig. 6C). Rats receiving the triple combination treatment with Org 24598 6 mg/kg, bupropion and varenicline presented a DA peak of approx. 60% that in contrast to combined treatment with bupropion and varenicline alone appeared to be sustained at a slightly higher level throughout the length of the experiment (140 min) (treatment effect *F*_(3,29)_ = 44.33, *p* < 0.0001, time effect *F*_(6, 174)_ = 1.15, *p* = 0.336, interaction term *F*_(18,174)_ = 7.36, *p* = 0.242, vehicle vs bupropion + varenicline *p* < 0.0001, vehicle vs Org 24598 + bupropion + varenicline *p* < 0.0001 (Fig. 6C). A two-way ANOVA did not reveal any significant differences between the two groups (Org 24598 + bupropion + varenicline vs bupropion + varenicline, *p* = 0.072, Fig. 6C) but upon comparisons of AUCs, a significantly higher DA output was evident for rats receiving the triple combination treatment as compared to bupropion + varenicline (treatment effect *F*_(3,29)_ = 36.17, *p* < 0.0001, vehicle vs bupropion + varenicline *p* < 0.0001, vehicle vs Org 24598 + bupropion + varenicline *p* < 0.0001, Org 24598 + bupropion + varenicline vs bupropion + varenicline, *p* = 0.0404, Fig. 6D).

## Discussion

Compromised dopaminergic neurotransmission, either resultant from innate variability in DA tone or acquired through chronic alcohol intake, has been associated with craving for, and consumption of alcohol in both rats and humans (Weiss et al. 1996; Heinz et al. 2004; Feltmann et al. 2016; Ericson et al. 2020). Accordingly, interventions that raise basal accumbal DA levels, thereby alleviating negative reinforcement to seek alcohol, might also reduce alcohol intake in rodents and in man (Koob and Volkow 2016; Soderpalm et al. 2017; Chau et al. 2018; Kranzler and Soyka 2018; Söderpalm et al. 2020). Combined use of pharmacotherapies that target the mesolimbic DA system in slightly different, yet complementary ways, may constitute a new pharmacological approach to combat AUD. Thereby, additive effects in counteracting a hypothesized mesolimbic hypodopaminergia may be achieved, that conceivably suppress alcohol intake while simultaneously enabling a reduction in drug dose and associated side effects. Indeed, a recent study in rats presented additive effects on accumbal DA levels and a synergistic ADE-abolishing action following combined administration of bupropion and varenicline, drugs that raise accumbal DA levels in two complementary ways (Söderpalm et al. 2020). Notably, a large body of evidence suggests that GlyR signaling is involved both in producing a DA elevation in response to alcohol intake and in maintaining the basal DA tone, pivotal for the positively and negatively reinforcing effects of alcohol intake, respectively (Molander and Soderpalm 2005a; Molander and Söderpalm 2005b). To further tailor a pharmacological treatment that targets how alcohol interacts with the reward system, this study therefore examined the effects of interfering with GlyRs in addition to nAChRs and DA- and noradrenaline-transporter proteins.

This study demonstrates that varenicline and a lower dose of bupropion than previously used for reducing alcohol intake in rats in combination with varenicline (Söderpalm et al. 2020), significantly abolishes the ADE when combined with Org 24598. Indeed, within-group comparisons (before and after the alcohol deprivation period) revealed ADE-reducing actions for all treatment groups, but when comparing the changes to that of the control group, only the triple combination produced a significant effect. Hence, the alcohol deprivation abolishing effect of bupropion + varenicline appears to be facilitated by Org 24598, which may be explained by its additive effect on accumbal DA levels observed in the present microdialysis study. It should be noted that the present study did not include a treatment arm with rats receiving a higher dose of bupropion + varenicline, and hence it is merely suggested but not ascertained that equal therapeutic effects can be achieved with a lower dose of bupropion combined with Org 24598 and varenicline. Further, in line with previous studies of other GlyT1-inhibitors, we show that also a low dose of Org 24598 raises extracellular levels of glycine (Lidö et al. 2009; Alberati et al. 2012; Hofmann et al. 2016) and that this dose appears to reduce intermittent alcohol intake. DA levels were not significantly raised following monotherapy with Org 24598, which is probably related to the low dose applied and/or a high frequency of DA non-responders. This phenomenon has been observed for several glycinergic treatments and may be related to differences in receptor subunit constellations and/or susceptibility to desensitization, or to baseline DA levels (Raltschev et al. 2016; Soderpalm et al. 2017; Olsson et al. 2020, 2022). Although not examined in the present study, GlyR desensitization resultant from GlyT1-treatment may also have mitigated the alcohol-induced DA response, as shown for local treatment with glycine itself and systemic treatment with another sarcosine-based GlyT1-inhibitor, possibly contributing both to the effect on intermittent alcohol intake and to the superior effect in attenuating the ADE by the triple combination treatment (Molander et al. 2005; Lidö et al. 2009).

Upon sufficient GlyR activation in nAc, either by positive allosteric modulation by alcohol or enhanced ligand pressure following Org 24598, accumbal DA levels are probably raised through GlyR-mediated disinhibition of tegmental dopaminergic firing by involving activation of nAChRs different from α4β2 nAChRs (Lynch, 2004, Burgos et al. 2015; Soderpalm et al. 2017). From recent studies it can be inferred that basal DA levels are probably upheld by alcohol-insensitive, heteromeric GlyRs located intrasynaptically on accumbal MSNs, whereas the alcohol-induced DA response instead involves alcohol-sensitive, homomeric alpha-1 and/or alpha2-containing GlyRs located extrasynaptically (Forstera et al. 2017, Soderpalm et al. 2017, Muñoz et al. 2018, San Martin et al. 2020). Varenicline is instead thought to act on α4β2 nAChRs present on tegmental DA neurons and regulating basal (Rollema et al. 2007) but not alcohol-induced DA release, *i.e.* presumably engaging similar but not identical downstream signaling pathways as glycine. Further, bupropion raises accumbal DA levels by inhibiting DA transporters (DAT) on DA terminals. Consequently, the triple combination treatment interferes with mesolimbic DA signaling in three complementary ways, which may enable the observed effects on ADE. Interestingly, basal accumbal DA release has previously been reported to be potentiated following reintroduction of bupropion after chronic administration and a short period of withdrawal, in line with the current study design for voluntary alcohol intake (Warner and Shoaib 2005). However, the current microdialysis study only entailed acute administration in a separate, non-alcohol exposed batch of rats which limits causal interpretations between DA response, alcohol intake and vice versa. Since chronic exposure to alcohol, withdrawal and repeated drug treatment may interfer with how the drugs are affecting the DA system, future studies may benefit from invoking monitoring of DA levels concomitantly with alcohol drinking behavior.

A few other shortcomings in the study should be discussed. In the alcohol consumption study, rats receiving Org 24598 presented signs of subacute, mild motor dysfunction which motivated a dose reduction starting on the third treatment day. It cannot be ruled out that these initial motor deficiencies contributed to attenuating the ADE for the triple combination treatment, but appears less likely since symptoms were mild, completely reversible, did not significantly alter water consumption and equally affected rats receiving monotherapy or combination treatment with Org 24598. Notably, no adverse symptoms were observed in the microdialysis studies, which may be explained by differences in treatment protocols (*i.e.* acute *vs.* repeated treatment) or that alcohol was not simultaneously on board. In another study in Wistar rats, repeated treatment with doses up to 16 mg/kg did not result in side effects motivating a dose reduction (Lidö et al. 2012). However, several rodent studies have described motor deficiencies ranging from none to intermittent and mild to continuous and severe, that are probably related to a high density of GlyRs in the rodent spinal cord and caudal brain as well as mode of inhibition and chemotype (*e.g.* sarcosine- or benzoylpiperazine-based) for the chosen GlyT-1 inhibitor (Le Pen et al. 2003; Kopec et al. 2010; Pinard et al. 2018; Cioffi 2021). Moreover, besides being an inhibitory GlyR agonist glycine is a co-agonist at NMDA-Rs, possibly increasing the risk for glutamatergic excitotoxicity following GlyT1-inhibition (Whitehead et al. 2004; Betz et al. 2006). When evaluating two sarcosine-based GlyT1-inhibitors in an animal model of pain, desired analgesic effects ascribed potentiated glycinergic signaling were delayed and preceded by paradoxical proalgesic effects that were abolished following pre-treatment with NMDA-R-inhibitors (Morita et al. 2008). Taken together, the propensity for rats to develop adverse effects from GlyT-1-inhibitors appears to vary between rat strains and batches and to rely on pharmacokinetic properties of the glycinergic agent. Consequently, future studies may benefit from using a non-sarcosine-based and/or reversible GlyT-1-inhibitor, successively increasing the dose and/or co-administrating an NMDA-R-antagonist. Importantly, in contrast to previous experiences in rodents, no signs of motor deficiencies or other major adverse effects have been identified in clinical trials evaluating different classes of GlyT-1-inhibitors or glycine itself (Harvey and Yee 2013; de Bejczy et al. 2014; Balu 2016; Hofmann et al. 2016). Accordingly, it is conceivable that also the triple combination treatment would be well-tolerated in humans.

To conclude, these results suggest that addition of a low dose of Org 24598 to varenicline and bupropion may allow for a dose reduction of bupropion while still maintaining the desired effect on the ADE, predictive of clinical efficacy in man. We argue that these effects presumably are mediated by enhancement of mesolimbic DA signaling through complementary mechanisms of action, since an additive DA response was observed in the present microdialysis study. As a next step, the triple combination treatment may be refined by *e.g.* further reducing the dose of bupropion or varenicline since also less pronounced alterations in basal DA levels may suffice to counteract hypodopaminergia and achieve ensuing attenuation in ADE. Moreover, exploration of combinatory treatments that employ reversible GlyT1-inhibitors, or other compounds tailored to specifically interfere with synaptic GlyR signaling or GlyR subunit constellations preferentially expressed in supratentorial regions, are warranted. Ultimately, combined targeting of mesolimbic GlyRs, DA reuptake proteins and nAChRs, may constitute a new pharmacological treatment principle for AUD that concomitantly interferes with the negative and positive reinforcing properties of alcohol.

### Supplementary Information

Below is the link to the electronic supplementary material.

Supplementary file1 (DOCX 123 kb)

## Data Availability

The datasets generated during and/or analyzed during the current study are available from the corresponding author on reasonable request.

## References

[CR1] Alberati D, Moreau JL, Lengyel J, Hauser N, Mory R, Borroni E, Pinard E, Knoflach F, Schlotterbeck G, Hainzl D, Wettstein JG (2012). Glycine reuptake inhibitor RG1678: a pharmacologic characterization of an investigational agent for the treatment of schizophrenia. Neuropharmacology.

[CR2] Anthenelli RM, Benowitz NL, West R, St Aubin L, Mcrae T, Lawrence D, Ascher J, Russ C, Krishen A, Evins AE (2016). Neuropsychiatric safety and efficacy of varenicline, bupropion, and nicotine patch in smokers with and without psychiatric disorders (EAGLES): a double-blind, randomised, placebo-controlled clinical trial. Lancet.

[CR3] Balu DT (2016). The NMDA receptor and schizophrenia: from pathophysiology to treatment. Adv Pharmacol.

[CR4] Betz H, Gomeza J, Armsen W, Scholze P, Eulenburg V (2006). Glycine transporters: essential regulators of synaptic transmission.

[CR5] Burgos CF, Muñoz B, Guzman L, Aguayo LG (2015). Ethanol effects on glycinergic transmission: from molecular pharmacology to behavior responses. Pharmacol Res.

[CR6] Chau P, Lido HH, Soderpalm B, Ericson M (2018). Acamprosate's ethanol intake-reducing effect is associated with its ability to increase dopamine. Pharmacol Biochem Behav.

[CR7] Cioffi CL (2021). Inhibition of glycine re-uptake: a potential approach for treating pain by augmenting glycine-mediated spinal neurotransmission and blunting central nociceptive signaling. Biomolecules.

[CR8] de Bejczy A, Nations KR, Szegedi A, Schoemaker J, Ruwe F, Soderpalm B (2014). Efficacy and safety of the glycine transporter-1 inhibitor org 25935 for the prevention of relapse in alcohol-dependent patients: a randomized, double-blind, placebo-controlled trial. Alcohol Clin Exp Res.

[CR9] de Bejczy A, Lof E, Walther L, Guterstam J, Hammarberg A, Asanovska G, Franck J, Isaksson A, Soderpalm B (2015). Varenicline for treatment of alcohol dependence: a randomized, placebo-controlled trial. Alcohol Clin Exp Res.

[CR10] di Chiara G, Imperato A (1988). Drugs abused by humans preferentially increase synaptic dopamine concentrations in the mesolimbic system of freely moving rats. Proc Natl Acad Sci.

[CR11] Ericson M, Molander A, Löf E, Engel JA, Söderpalm B (2003). Ethanol elevates accumbal dopamine levels via indirect activation of ventral tegmental nicotinic acetylcholine receptors. Eur J Pharmacol.

[CR12] Ericson M, Ulenius L, Andrén A, Jonsson S, Adermark L, Söderpalm B (2020). Different dopamine tone in ethanol high- and low-consuming Wistar rats. Addict Biol.

[CR13] Feltmann K, Fredriksson I, Wirf M, Schilström B, Steensland P (2016). The monoamine stabilizer (-)-OSU6162 counteracts downregulated dopamine output in the nucleus accumbens of long-term drinking Wistar rats. Addict Biol.

[CR14] Forstera B, Munoz B, Lobo MK, Chandra R, Lovinger DM, Aguayo LG (2017). Presence of ethanol-sensitive glycine receptors in medium spiny neurons in the mouse nucleus accumbens. J Physiol.

[CR15] Hansson AC, Gründer G, Hirth N, Noori HR, Spanagel R, Sommer WH (2019). Dopamine and opioid systems adaptation in alcoholism revisited: convergent evidence from positron emission tomography and postmortem studies. Neurosci Biobehav Rev.

[CR16] Harvey RJ, Yee BK (2013). Glycine transporters as novel therapeutic targets in schizophrenia, alcohol dependence and pain. Nat Rev Drug Discov.

[CR17] Hasin DS, O’Brien CP, Auriacombe M, Borges G, Bucholz K, Budney A, Compton WM, Crowley T, Ling W, Petry NM (2013). DSM-5 criteria for substance use disorders: recommendations and rationale. Am J Psychiatry.

[CR18] Heinz A, Siessmeier T, Wrase J, Hermann D, Klein S, Grüsser SM, Flor H, Braus DF, Buchholz HG, Gründer G, Schreckenberger M, Smolka MN, Rösch F, Mann K, Bartenstein P (2004). Correlation between dopamine D(2) receptors in the ventral striatum and central processing of alcohol cues and craving. Am J Psychiatry.

[CR19] Hofmann C, Pizzagalli F, Boetsch C, Alberati D, Ereshefsky L, Jhee S, Patat A, Boutouyrie-Dumont B, Martin-Facklam M (2016). Effects of the glycine reuptake inhibitors bitopertin and RG7118 on glycine in cerebrospinal fluid: results of two proofs of mechanism studies in healthy volunteers. Psychopharmacology.

[CR20] Howard EC, Schier CJ, Wetzel JS, Gonzales RA (2009). The dopamine response in the nucleus accumbens core-shell border differs from that in the core and shell during operant ethanol self-administration. Alcohol Clin Exp Res.

[CR21] Jonas DE, Amick HR, Feltner C, Bobashev G, Thomas K, Wines R, Kim MM, Shanahan E, Gass CE, Rowe CJ, Garbutt JC (2014). Pharmacotherapy for adults with alcohol use disorders in outpatient settings: a systematic review and meta-analysis. JAMA.

[CR22] Konar MSG, Zeilhofer HU, Aguayo LG (2022). Structural evidence of the origin of glycinergic innervation in nucleus accumbens.

[CR23] Koob GF, Volkow ND (2016). Neurobiology of addiction: a neurocircuitry analysis. The Lancet Psychiatry.

[CR24] Kopec K, Flood DG, Gasior M, McKenna BA, Zuvich E, Schreiber J, Salvino JM, Durkin JT, Ator MA, Marino MJ (2010). Glycine transporter (GlyT1) inhibitors with reduced residence time increase prepulse inhibition without inducing hyperlocomotion in DBA/2 mice. Biochem Pharmacol.

[CR25] Kranzler HR, Soyka M (2018). Diagnosis and pharmacotherapy of alcohol use disorder: a review. JAMA.

[CR26] Larsson A, Edström L, Svensson L, Söderpalm B, Engel JA (2005). Voluntary ethanol intake increases extracellular acetylcholine levels in the ventral tegmental area in the rat. Alcohol Alcohol.

[CR27] le Pen G, Kew J, Alberati D, Borroni E, Heitz MP, Moreau JL (2003). Prepulse inhibition deficits of the startle reflex in neonatal ventral hippocampal-lesioned rats: reversal by glycine and a glycine transporter inhibitor. Biol Psychiatry.

[CR28] Lidö HH, Stomberg R, Fagerberg A, Ericson M, Söderpalm B (2009). The glycine reuptake inhibitor org 25935 interacts with basal and ethanol-induced dopamine release in rat nucleus accumbens. Alcohol Clin Exp Res.

[CR29] Lidö HH, Marston H, Ericson M, Söderpalm B (2012). The glycine reuptake inhibitor Org24598 and acamprosate reduce ethanol intake in the rat; tolerance development to acamprosate but not to Org24598. Addict Biol.

[CR30] Litten RZ, Ryan ML, Fertig JB, Falk DE, Johnson B, Dunn KE, Green AI, Pettinati HM, Ciraulo DA, Sarid-Segal O, Kampman K, Brunette MF, Strain EC, Tiouririne NA, Ransom J, Scott C, Stout R (2013). A double-blind, placebo-controlled trial assessing the efficacy of varenicline tartrate for alcohol dependence. J Addict Med.

[CR31] Loftén A, Adermark L, Ericson M, Söderpalm B (2021). An acetylcholine-dopamine interaction in the nucleus accumbens and its involvement in ethanol's dopamine-releasing effect. Addict Biol.

[CR32] Lynch JW (2004). Molecular structure and function of the glycine receptor chloride channel. Physiol Rev.

[CR33] Maisel NC, Blodgett JC, Wilbourne PL, Humphreys K, Finney JW (2013). Meta-analysis of naltrexone and acamprosate for treating alcohol use disorders: when are these medications most helpful?. Addiction.

[CR34] McKee SA, Harrison EL, O'Malley SS, Krishnan-Sarin S, Shi J, Tetrault JM, Picciotto MR, Petrakis IL, Estevez N, Balchunas E (2009). Varenicline reduces alcohol self-administration in heavy-drinking smokers. Biol Psychiatry.

[CR35] Miller CN, Kamens HM (2020). The role of nicotinic acetylcholine receptors in alcohol-related behaviors. Brain Res Bull.

[CR36] Molander A, Soderpalm B (2005). Glycine receptors regulate dopamine release in the rat nucleus accumbens. Alcohol Clin Exp Res.

[CR37] Molander A, Söderpalm B (2005). Accumbal strychnine-sensitive glycine receptors: an access point for ethanol to the brain reward system. Alcohol Clin Exp Res.

[CR38] Molander A, Lof E, Stomberg R, Ericson M, Soderpalm B (2005). Involvement of accumbal glycine receptors in the regulation of voluntary ethanol intake in the rat. Alcohol Clin Exp Res.

[CR39] Molander A, Lidö HH, Löf E, Ericson M, Söderpalm B (2007). The glycine reuptake inhibitor Org 25935 decreases ethanol intake and preference in male wistar rats. Alcohol Alcohol.

[CR40] Morita K, Motoyama N, Kitayama T, Morioka N, Kifune K, Dohi T (2008). Spinal antiallodynia action of glycine transporter inhibitors in neuropathic pain models in mice. J Pharmacol Exp Ther.

[CR41] Muñoz B, Yevenes GE, Förstera B, Lovinger DM, Aguayo LG (2018). Presence of inhibitory glycinergic transmission in medium spiny neurons in the nucleus accumbens. Front Mol Neurosci.

[CR42] Olsson Y, Höifödt Lidö H, Danielsson K, Ericson M, Söderpalm B (2020). Effects of systemic glycine on accumbal glycine and dopamine levels and ethanol intake in male Wistar rats. J Neural Transm.

[CR43] Olsson Y, Lidö H, Ericson M, Söderpalm B (2022). The glycine-containing dipeptide leucine-glycine raises accumbal dopamine levels in a subpopulation of rats presenting a lower endogenous dopamine tone. J Neural Transm.

[CR44] Pinard E, Borroni E, Koerner A, Umbricht D, Alberati D (2018). Glycine transporter type I (GlyT1) inhibitor, bitopertin: a journey from lab to patient. Chimia.

[CR45] Raltschev C, Hetsch F, Winkelmann A, Meier JC, Semtner M (2016). Electrophysiological signature of homomeric and heteromeric glycine receptor channels. J Biol Chem.

[CR46] Rehm J, Gmel GE, Gmel G, Hasan OSM, Imtiaz S, Popova S, Probst C, Roerecke M, Room R, Samokhvalov AV, Shield KD, Shuper PA (2017). The relationship between different dimensions of alcohol use and the burden of disease-an update. Addiction.

[CR47] Rollema H, Chambers LK, Coe JW, Glowa J, Hurst RS, Lebel LA, Lu Y, Mansbach RS, Mather RJ, Rovetti CC, Sands SB, Schaeffer E, Schulz DW, Tingley FD, WILLIAMS KE (2007). Pharmacological profile of the alpha4beta2 nicotinic acetylcholine receptor partial agonist varenicline, an effective smoking cessation aid. Neuropharmacology.

[CR48] San Martin L, Gallegos S, Araya A, Romero N, Morelli G, Comhair J, Harvey RJ, Rigo JM, Brone B, Aguayo LG (2020). Ethanol consumption and sedation are altered in mice lacking the glycine receptor α2 subunit. British J Pharmacol.

[CR49] Soderpalm B, Lido HH, Ericson M (2017). The glycine receptor-A functionally important primary brain target of ethanol. Alcohol Clin Exp Res.

[CR50] Söderpalm B, Danielsson K, de Bejczy A, Adermark L, Ericson M (2020). Combined administration of varenicline and bupropion produces additive effects on accumbal dopamine and abolishes the alcohol deprivation effect in rats. Addict Biol.

[CR51] Spanagel R (2009). Alcoholism: a systems approach from molecular physiology to addictive behavior. Physiol Rev.

[CR52] Steensland P, Simms JA, Holgate J, Richards JK, Bartlett SE (2007). Varenicline, an alpha4beta2 nicotinic acetylcholine receptor partial agonist, selectively decreases ethanol consumption and seeking. Proc Natl Acad Sci U S A.

[CR53] Ulenius L, Andrén A, Adermark L, Söderpalm B, Ericson M (2020). Sub-chronic taurine administration induces behavioral sensitization but does not influence ethanol-induced dopamine release in the nucleus accumbens. Pharmacol Biochem Behav.

[CR54] Vena AA, Zandy SL, Cofresí RU, Gonzales RA (2020). Behavioral, neurobiological, and neurochemical mechanisms of ethanol self-administration: a translational review. Pharmacol Ther.

[CR55] Vengeliene V, Rossmanith M, Takahashi TT, Alberati D, Behl B, Bespalov A, Spanagel R (2018). Targeting glycine reuptake in alcohol seeking and relapse. J Pharmacol Exp Ther.

[CR56] Warner C, Shoaib M (2005). How does bupropion work as a smoking cessation aid?. Addict Biol.

[CR57] Weiss F, Parsons LH, Schulteis G, Hyytiä P, Lorang MT, Bloom FE, Koob GF (1996). Ethanol self-administration restores withdrawal-associated deficiencies in accumbal dopamine and 5-hydroxytryptamine release in dependent rats. J Neurosci.

[CR58] Whitehead KJ, Pearce SM, Walker G, Sundaram H, Hill D, Bowery NG (2004). Positive N-methyl-D-aspartate receptor modulation by selective glycine transporter-1 inhibition in the rat dorsal spinal cord in vivo. Neuroscience.

