# Supplementary material for: The GlyT1-inhibitor Org 24598 facilitates the alcohol deprivation abolishing and dopamine elevating effects of bupropion + varenicline in rats
Source: J Neural Transm (Vienna). 2023 Sep 29;131(1):95–106. doi: 10.1007/s00702-023-02701-x (PMC10769923; doi:10.1007/s00702-023-02701-x)
Supplement: Supplementary file 1 — Supplementary file1 (DOCX 123 kb) [file 702_2023_2701_MOESM1_ESM.docx]

**Supplementary figure 1.**


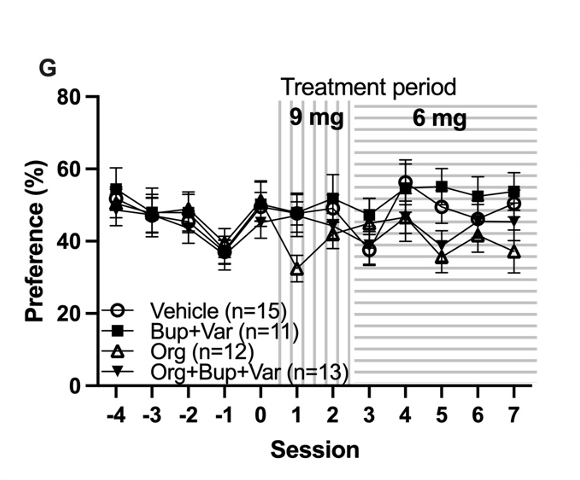


**Supplementary figure 1. Effects of Org 24598 6 and 9 mg/kg, bupropion and varenicline, alone or combined on preference for alcohol.** Alcohol preference over time following seven days of treatment with vehicle, bupropion+varenicline or Org 24598 alone or combined with bupropion+varenicline was not significantly altered. Rats treated with Org 24598 received a higher dose (9 mg/kg) during the first two days, whereafter it was reduced to 6 mg/kg for the remaining five days of active treatment. During baseline and the treatment period, rats had limited access to alcohol (8h-sessions/day). Shown are mean values ± SEM, n=number of rats.
